# Light-dark cycle synchronization of circadian rhythm in blind primates

**DOI:** 10.1186/1740-3391-3-10

**Published:** 2005-09-06

**Authors:** Mayara MA Silva, Alex M Albuquerque, John F Araujo

**Affiliations:** 1 Laboratório de Cronobiologia, Departamento de Fisiologia, CB/UFRN, Natal, Brazil

## Abstract

**Background:**

Recently, several papers have shown that a small subset of retinal ganglion cells (RGCs), which project to the suprachiasmatic nucleus (SCN) and contain a new photopigment called melanopsin, are the photoreceptors involved in light-dark entrainment in rodents. In our primate colony, we found a couple of common marmosets (*Callithrix jacchus*) that had developed progressive and spontaneous visual deficiency, most likely because of retinal degeneration of cones and/or rods. In this study, we evaluated the photoresponsiveness of the circadian system of these blind marmosets.

**Methods:**

Two blind and two normal marmosets were kept in cages with a controlled light-dark cycle (LD) to study photoentrainment, masking, and phase response to a dark pulse.

**Results:**

Blind marmosets were entrained with the new LD cycle when light onsets were delayed and advanced by 6 hours. In constant light conditions, blind marmosets free-ran with a period of 23.2 hours, while normal animals free-ran with a period of 23.6 hours. All marmosets responded to dark pulses in the early subjective day with phase delays and with phase advances in the late subjective day.

**Conclusion:**

Our results demonstrate that light can synchronize circadian rhythms of blind marmosets and consequently, that this species could be a good primate model for circadian photoreception studies.

## Introduction

In mammals, circadian rhythms of physiological and behavioral variables are driven by a master circadian pacemaker, the suprachiasmatic nucleus (SCN). To be useful, the circadian clock must be synchronized to the light and dark alternation of the real world's day-night cycles. In mammals, the eyes are required for photoentrainment. Because only rods and cones were known to be ocular photoreceptors, it was generally assumed that circadian photoreception relied on these cells. However, studies on *rd*/*rd *mice, which lack rod photoreceptors, and more recent studies on *rd*/*rd cl *mice, which lack all functional rods and cones, have provided overwhelming evidence that these classical photoreceptors are not required for photoentrainment [1,2]. Recently, several papers have shown that a small subset of retinal ganglion cells (RGCs) that project to the SCN and contain a new photopigment called melanopsin serve as photoreceptors involved in light-dark entrainment in rodents [3-7]. Melanopsin was also found in humans, other primates, rats, and mice [8]. Several studies have shown that the melanopsin photoreceptors not only regulate the circadian system, but also contribute to both papillary light reflex and acute alterations in motor activity, and may be involved in a broad range of physiological and behavioral responses to light [8].

The common marmoset (*Callithrix jacchus*) is a small neo-tropical primate found in the northeast of Brazil and is easily adapted to laboratory use. It is a diurnal animal and has a bimodal circadian pattern. The motor activity of these animals displays a stable circadian rhythm in constant light. Light pulses cause delays when given in the early subjective night and phase advances when given in the late subjective night [9,10]. Studies of morphology of retinal ganglion cells in marmosets have shown a sex-linked polymorphism of cone pigment expression, such that all males are dichromats and the majority of females are trichromats [11].

In our primate colony, we found a couple of common marmosets that had developed progressive and spontaneous visual deficiency. They were living in semi-natural conditions and were active during the day and inactive at night. Ophthalmoscopic examination did not show opacities of the cornea, lens, and vitreous, nor lesions of the optic nerve, nor vascular retinopathies. The most important feature in fundoscopy was a bilateral pigmentation in the macular region, which is similar to retinal rod degeneration. As it is known that ganglion cells are generally preserved in retinal degeneration disease [12], we proposed that these marmosets have a retinal degeneration of cones and/or rods. Based on previous studies, we believe that this retinal degeneration of rods and cones does not impair expression of circadian rhythmicity, photoentrainment, masking, and phase response to a dark pulse. In order to test this hypothesis, these marmosets were transferred to the laboratory so that the light-dependent features of their circadian system could be studied.

## Methods

Four marmosets (one normal male, one blind male, one normal female, and one blind female), with an average age of eight years and average weight of 354 g, were housed in individual cages in a room with attenuated noise, controlled temperature (average temperature of 25.5°C), water *at libitum *and food daily available for 8.5 hours. The animals were first exposed to an LD cycle of 24 hours (LD 12:12). Illuminance was 150 lux during the light phase and 1 lux during the dark phase. The marmosets were adapted to the laboratory for 10 days. After two weeks in LD 12:12, the time of lights-on was delayed by 6 hours; three weeks later, it was advanced by 6 hours. Four weeks later, the marmosets were placed in constant light conditions for 4 weeks. The marmosets were then returned to LD 12:12 for 3 more weeks and then again to constant light for 4 weeks.

General circadian locomotor activity of the marmosets was measured using an infrared motion sensor above the cage. Output from the sensors was integrated with an IBM-compatible computer running data acquisition software. Analyses of rhythm characteristics and graphical output, actograms, were undertaken using the El Temps computer program (Diez-Noguera, Barcelona, Spain). The free-running period of the locomotor activity rhythm under constant light was computed by the chi square periodogram procedure [13] with a global risk level (α) of *p *< 0.05. Under LL, the onset of activity, designated as circadian time (CT) 0, was used as the phase reference point for the onset of the subjective day. Phase-shifts were determined as the difference between projected times of activity onset on the day after dark stimulation. The dark stimulation consisted of 2-hour pulses of darkness. Experiments were in compliance with the institutional guidelines of the Universidade Federal do Rio Grande do Norte and Sociedade Brasileira de Neurociência e Comportamento.

## Results and discussion

The marmosets were submitted to two behavioral tests. In the first one, a non-smelling object (such as a pen or a key) was placed two centimeters away from each animal's face. The normal marmosets directed their sight to the object and tried to grab and bite it; the blind animals did not react to the objects at all. The same test was repeated with the objects in movement, and again only the normal marmosets reacted by directing their sight to the moving object. The second test took place in a room with dim light (10 lux). A spotlight was placed on one side of the animals and directed to their faces. The normal marmosets turned their faces towards the light, while the blind ones did not.

**Figure 1:**
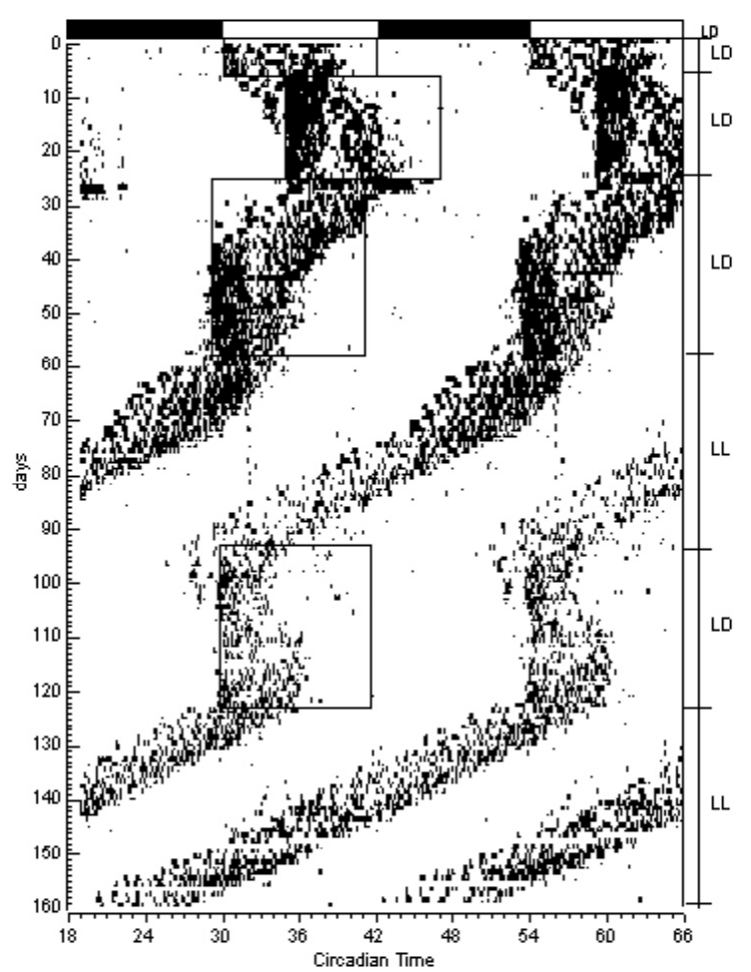
**Light-dark cycle synchronization of circadian rhythms in blind primates**. Shown are representative double-plotted motor activity records of a blind marmoset during photoentrainment, advance and delay of light phase, and free-run in constant light condition (LL). Time is indicated at the bottom, day at left side, and the light-dark cycle (LD) or LL on the right side. The boxes represent the light phase of the LD cycle.

**Figure 2:**
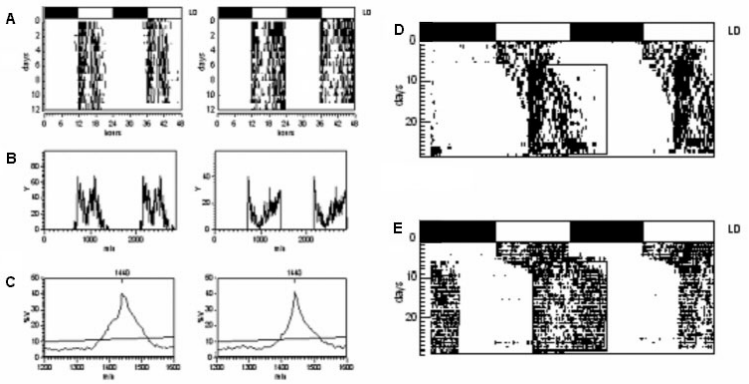
**Circadian pattern of motor activity in blind and normal marmoset**. (A) Representative double-plotted motor activity of a blind (left) and a normal (right) animal. (B) Wave form plot of activity of blind (left) and normal (right) marmoset. Both animals showed a bimodal pattern of activity, but this bimodal pattern was less prominent in the blind animal, which showed a shorter active phase compared to the normal animal. (C) Periodogram of activity rhythms for blind (left) and normal (right) marmoset in the LD cycle. (D) Entrainment of the blind marmoset after the time of lights-on was delayed by 6 hours. (E) Entrainment of the sighted marmoset after the time of lights-on was delayed by 6 hours.

As shown in Figure 1, blind marmosets were clearly synchronized to the external cycles. Like normal animals, blind animals showed a normal biphasic activity circadian rhythm, with a more intense bout of activity at the beginning of the light phase and a second bout near the end. However, this bimodal pattern was less prominent in blind animals (Figure 2). Additionally, blind marmosets showed a shorter active phase compared to normal animals. After we shifted the light phase by 6 hours (first a delay and then an advance), blind marmosets were entrained to the new light-dark cycle, but their entrainment was much slower compared to the normal marmosets (Figures 1 and 2). The blind animals synchronized only after 12–14 days, while the normal animals did so after 3–4 days. During entrainment, the phase angle of activity onset in relation to the LD cycle was different in blind and normal marmosets (see Figure 2).

The animals were then placed in constant light conditions in order to determine if they had functional circadian clocks. As show in Figure 1, the marmosets showed free-running circadian rhythms. Free-running periods were significantly different between the two groups: blind marmosets showed a 23.2-hour period while normal marmosets free-ran showed a period of 23.6 hours. This shorter period in blind marmosets could be explained by their lower activity and, consequently, decreased motor activity feedback to the circadian system, or by the participation of classical photoreceptors (rods and cones) in the generation of free-running circadian rhythms. However, it could also be explained by the suggestion that the loss of rods and cones has an impact on the nature of light information reaching the SCN [8].

For photic entrainment to occur, the circadian oscillator must respond differently to light at different phases of its cycle. Phase response curves (PRC) are useful descriptions of these phase-dependent responses. A number of non-photic stimuli, both pharmacological and non-pharmacological, have been identified as able to induce phase shifts in mammalian circadian clocks as a function of the circadian phase that the stimulus is presented [14]. The PRC of non-photic stimuli (including dark pulses presented to animals kept in constant light) is 180° out of phase with photic stimuli. We tested the phase-shift response of blind marmosets using dark pulses of 2-hour duration. When the dark pulse was given in the early subjective day, it caused a phase delay; when given in the late subjective day, it caused a phase advance. This result is an important contribution to the discussion about the non-photic phase shift in this species. Our results agree with Glass et al [14], in whose studies the qualitative similarities between the phase responses to entraining photic and non-photic stimuli in marmosets and nocturnal mammals were demonstrated.

Many of the non-photic stimuli that induce phase shifts in the circadian clocks also induce an acute increase in locomotor activity in nocturnal mammals, and it appears that at least some of the phase shifting effects of these agents is due to the induction of activity and/or arousal [15]. In the present study in marmosets, the phase shifts produced by dark pulses were not due to the inhibition of activity. The dark pulses produced an inhibition of activity (negative masking) in the sighted marmosets but not in the blind ones, despite the fact that both groups showed phase shifts with dark pulses.

The response of the circadian system to different stimuli, photic and non-photic, is of great importance because implies that circadian systems are in fact able to use many sources of information. As the marmoset is a social animal, we also investigated social synchronization between these animals. Blind marmosets showed different activity onset during the free-running phase, but they showed a stable phase angle. The two normal marmosets showed the same behavior but with different free-running periods from the blind marmosets. Therefore, despite the fact that the four animals were in the same room, the blind marmosets were not synchronized with the normal ones.

One limitation of this study is the small number of animals, but the results of the two normal marmosets are similar to other studies that were conducted in our laboratory [16]. Considering studies previously carried out in rodents along with our present results, it is possible to infer that the blind marmosets had normal retinal ganglion cells, which are required to synchronize their circadian clocks to the LD cycle. In the absence of classical photoreceptors, photosensitive ganglion cells are sufficient for photic entrainment [17].

## Conclusion

Ours results constitute the first experimental evidence that non-classical retinal photoreceptors can provide photic information to the circadian system of primates and diurnal animals. The blind marmosets may provide an excellent model for the study of photoreception and entrainment in primates. Possible benefits are obvious, such as the development of strategies to solve the problem of synchronization in blind humans or to study retinal degeneration.

## Competing interests

The author(s) declare that they have no competing interests.

## Authors' contributions

MMAS: Participated in all experiments, in the analysis and discussion of the results, and in the writing of the manuscript.

AMA: Participated in all experiments, in the analysis and discussion of the results, and in the writing of the manuscript.

JFA: Participated in all experiments, in the analysis and discussion of the results, and in the writing of the manuscript.

All authors read and approved the final manuscript.
